# Attempts to restore loss of function in damaged ACD cells open the way to non-mutational oncogenesis

**DOI:** 10.1016/j.gendis.2023.101109

**Published:** 2023-09-12

**Authors:** Vladimir F. Niculescu

*This correspondence to Gene & Diseases calls attention to the controversy between proponents of genetic modification in carcinogenesis and supporters of non-mutational oncogenesis. Data from molecular cancer research and findings from evolutionary cancer cell biology support the latter claim and provide no rationale for the mutational origin of sporadic cancers. It is more conceivable that the general susceptibility of asymmetric cell division (ACD) phenotypes to oxygen leads to irreparable DNA defects and dysregulated defective symmetric cell division (DSCD) phenotypes, and their repair through an ancient* *polyploid* *MGRS (multinucleated giant repair structures) repair mechanism initiates oncogenesis. This is reported in detail in the following lines.*

I am a cell biologist working in the field of evolutionary cancer cell biology. My specialty is the evolutionary origin of the cancer genome and the non-mutational reprogramming of dysregulated cells to generate cancer stem cells (naive CSCs). Over the past 15 years, I have published numerous papers in this area. I have always strived to show that polyploid MGRS cancer is not a genetic disease caused by mutation, but arises from an extraordinary genome repair mechanism inherited from the Urgermline of the common ancestor of amoebozoans, metazoans, and fungi (AMF) that paves the way for oncogenesis.

In correspondence with your journal regarding my recent article “Cancer genes and cancer stem cells in tumorigenesis: Evolutionary deep homology and controversies”.^1^ Ivanovic and Vlaski-Lafarge write: “The ancient tool kit needed for the *stemness expression* is present in cancer but does not provoke cancer by itself. Cancer is induced when a *genetic alteration* results in dysregulation” (https://doi.org/10.1016/j.gendis.2023.01.003).

According to the current understanding, the ACD path rises to one differentiated cell and one self-renewing stem cell. In the symmetric cell division (SCD) path, one stem cell gives rise to either two differentiated cells or two undifferentiated stem cells. However, this is not always the case. As observed in the G + S life cycle of cancer and amoebae,^2^ a hyperoxic switch leads to the formation of two defective daughter cells that have completely lost their stemness and ACD potential (loss of function) but continue to proliferate through defective symmetric cell division (DSCD). These DSCD cells are no longer stem cells and cannot become stem cells again. However, they do not become apoptotic, remain viable, and can proliferate by defective symmetric cells.^2^ Homologous recombination repair is deficient, and the DNA defects are inherited by both daughter cells. The DSCD/homologous recombination deficiency phenotype of amoebae also occurs in humans, metazoans, and cancer. In the present paper, such defective cells that lose their function are referred to as DSCD cells. DSCD cells are not senescent cells with permanent cell cycle arrest and the prospect of cell death.

In many tissues of humans and mammalians, including the brain, loss of ACD function is associated with cancer. Disruption of ACD conditions inside or outside the niche leads to the devastating consequences of malignancy.^3^ Cancer risk is inherent to stressed stem cells, and this risk is associated with the ancestral gene regulatory network conserved in the genome of humans.^2^ The type of primary tumor depends on the type of the DSCD cells, its stem cell origin, and its niche of origin.

Older brain tumor studies provided evidence that CSCs arise from the inability of cells such as neural stem cells to divide asymmetrically, resulting in continuous symmetric mitosis (doi: 10.1016/j.cell.2009.06.048). The researchers hypothesize that an abnormal increase in symmetric cell divisions contributes to adopting a CSC phenotype. Today, we know that transforming cells are not normal stem cells but deregulated DSCDs without stemness and ACD potential. Loss of function is due to DNA damage and homologous recombination deficiency, and transformation does not occur by individual stimuli but rather by environmental changes in the niche.

Under normal conditions, ACD is the most common division, but this type of division occurs least frequently during chemotherapeutic exposure such as treatment with temozolomide.^3^ In exchange, symmetric SCD is the most frequently observed cell division. Temozolomide is a chemotherapeutic agent commonly used to treat brain tumors such as glioblastoma and anaplastic astrocytoma. It switches normal CSCs and progenitor cells to a DSCD phenotype similar to the DSCD cell type observed in amoebae. Unfortunately, researchers have not pursued the further development of these DSCD cells^3^

DSCD cells mostly remain in niches of temporary quiescence or niches of very slow-cycling (non-malignant niches). However, when they switch niches (or the niche itself changes), enhanced symmetric proliferation forms a high-density DSCD cell pool capable of fusing and forming a syncytium for genome repair and reprogramming. Such ancestral multinucleated giant repair structures (MGRS) have been observed in amoebae, but also in cancer (polyploid giant cancer cells or PGCCs).^2^ The individual nuclei of the syncytium fuse and form a giant polyploid nucleus capable of DNA damage repair and genome reprogramming. The MGRS pathway and its gene regulatory network are parts of the old AMF life cycle program conserved in the genome of humans and metazoans. Unfortunately, the ancient gene regulatory network drives the dysfunctional DSCDs into an oncogenic transformation process resulting in the formation of naive primary CSCs rather than back to normal human stem cells. Recent glioma studies demonstrate the emergence of *glioma stem cells from quiescent or slowly cycling non-cancerous stem cells* (https://doi.org/10.1155/2011/396076).

The fundamental mechanism by which the dysfunctional DSCD cells acquire cancer stemness and genomic integrity occurs in the absence of mutations and is somewhat consistent with hierarchical and CSC models,^4^ which assume that oncogenesis begins with stem cells that escape standard ACD growth control and regulation to form cells that can transform into CSCs. According to recent findings,^2^ primary naive CSCs differentiate a non-gametogenic cancer germline strongly related to the Urgermline and whose G + S life cycle. Both form the cell biological basis of tumors. The microenvironment of all intermediate stages and niches is essential for oncogenic transformation. The components and structure of the niche may vary depending on the organism and tissue.

There is evidence that the reprogramming life-cycle reversal niche is an oncogenic perivascular niche (PVN) but details on how these niches form are limited. The concept of a CSC/PVN was first described by Calabrese et al in 2007 (doi: 10.1016/j.ccr.2006.11.020) in the context of glioblastoma brain tumors is a highly vascularized cancer form. As recently reported by Akindona et al (doi: 10.3389/fonc.2022.947634), glioblastoma/PVNs include much more cell types than just CSCs, namely pericytes, reactive astrocytes, tumor-associated macrophages, microglia, and fibroblasts are other components that contribute to the maintenance of the glioblastoma CSC PVN. There are good reasons to assume that the oncogenic life-cycle reversal niche will develop a similar oxygenic range as the oncogenic PVN.

As early as five years ago, McGrail et al demonstrated that spontaneous DNA damage due to “oncogene-induced” replication stress is ubiquitous in precancerous cells, and that defects in the DNA replication stress response allow cells to proliferate further, ultimately leading to early tumorigenesis.^5^ According to the study, replication stress response defects cause non-malignant cells to transition to a cancer stem cell-like state. The researchers assumed that normal stem cells may have an innate ability to suppress the replication stress response. Recent work^2^ confirms this view but notes that the transformed cells are even homologous recombination deficiency cells and that the “innate ability” is a consequence of the oxygen sensitivity of all human and metazoan stem cell lines and non-gametogenic germlines, driven and controlled by the conserved ancestral gene regulatory network.^1^^,^^2^

In summary, although self-renewal and differentiation are fundamental properties of all ACD stem cells, this is only true as long as ACD cells are protected from harmful environmental conditions. If environmental conditions change or stem cells leave the niche and proliferate under hyperoxic conditions with an O_2_ content of more than 6.0%, they irrevocably lose their stemness and differentiation function and become DSCD cells. Attempting to return to the former properties, DSCD cells undergo genome reprogramming and oncogenic transformation.

## Conflict of interests

The author confirms that there is no conflict of interests.
